# Associations of activities of daily living and their trajectories with the risk of diabetes-related lower-limb amputation: evidence from the HRS and ELSA longitudinal cohorts

**DOI:** 10.3389/fendo.2026.1874068

**Published:** 2026-07-15

**Authors:** Hui Guo, Zunwang Li, Yunhui Zhang, Ruizheng Zhu, Zhihong Fu, Dongxiao Li, Junde Wu, Zhaojun Chen

**Affiliations:** Department of Orthopedics, Beijing University of Chinese Medicine Third Affiliated Hospital, Beijing, China

**Keywords:** activities of daily living, ADL, diabetes-related lower-limb amputation, DLLA, ELSA, HRS, older adults, trajectory analysis

## Abstract

**Background:**

Diabetes-related lower-limb amputation (DLLA) is a severe complication of diabetes associated with considerable disability and mortality. However, the associations of activities of daily living (ADL) and their longitudinal trajectories with DLLA risk remain unclear.

**Methods:**

Data from the Health and Retirement Study (HRS; n = 3007) and the English Longitudinal Study of Ageing (ELSA; n = 833) were analyzed. ADL trajectories were identified using Group-Based Trajectory Modeling. Associations of ADL levels and trajectories with diabetes-related lower-limb amputation (DLLA) were evaluated using Cox regression models, with competing-risk, nonlinear, subgroup, and mediation analyses performed as sensitivity and exploratory analyses.

**Results:**

During a median follow-up of approximately two survey waves (approximately four years), 986 DLLA events occurred in the HRS cohort and 254 events occurred in the ELSA cohort. Multivariable Cox regression analyses demonstrated that ADL scores were significantly associated with DLLA risk after adjustment for potential confounders (HRS: adjusted HR = 1.10, 95% CI: 1.07–1.14; ELSA: adjusted HR = 1.20, 95% CI: 1.12–1.28). Compared with the Stable-low trajectory group, the risks of DLLA were significantly higher in both the Stable-rise and Stable-high groups. The risk increased by approximately 51%–84% in the Stable-rise group and by 36%–1.31-fold in the Stable-high group. Kaplan–Meier survival analysis showed significant differences in DLLA incidence among the ADL trajectory groups (log-rank P < 0.001). The competing risk analyses yielded results consistent with those of the Cox models. RCS analysis indicated a significant nonlinear association between ADL and DLLA risk, with a threshold effect observed around ADL ≈ 2. Furthermore, mediation analysis suggested that depression partially mediated the association between ADL and DLLA risk.

**Conclusions:**

ADL levels and their longitudinal trajectories were significantly associated with the risk of DLLA among individuals with diabetes. Individuals with persistently poor functional status or progressively declining function exhibited the highest risk. Dynamic changes in functional status may serve as useful indicators for identifying individuals at elevated risk of DLLA. Routine assessment of ADL may contribute to risk stratification and clinical monitoring in individuals with diabetes.

## 1 Introduction

Diabetes represents one of the most pressing public health challenges worldwide, and its prevalence continues to increase with population aging and rapid urbanization. It has been estimated that in 2021 the global prevalence of diabetes among adults aged 20–79 years was 10.5% (536.6 million individuals), and this figure is projected to increase to 12.2% (783.2 million) by 2045 (1). More recent estimates indicate that approximately 589 million adults were living with diabetes in 2024, representing 11.1% of the global adult population, and this number is expected to reach 853 million by 2050 (2, 3). This growing burden disproportionately affects middle-aged and older adults. The combined effects of population aging and the rising prevalence of chronic diseases substantially increase their risk of developing diabetes-related complications.

Among the chronic complications of diabetes, those affecting the lower limbs—including diabetic foot ulcers, infection, gangrene, and lower-limb amputation—are particularly severe. Lower-limb amputation represents the most advanced and devastating stage of diabetes-related lower-limb disease and is associated with substantial morbidity, including severe physical disability, recurrent ulceration, and markedly increased mortality risk (4). The five-year mortality rate among individuals with diabetes-related foot disease is comparable to that observed for many common cancers, and the occurrence of lower-limb amputation further worsens long-term prognosis (5). Because detailed information on non-amputation lower-limb complications was not consistently available across the HRS and ELSA cohorts, the present study focused specifically on diabetes-related lower-limb amputation (DLLA) as the primary outcome. Beyond these clinical consequences, DLLA substantially impairs quality of life, reduces functional independence, and imposes significant socioeconomic burdens. These burdens arise from prolonged healthcare utilization, productivity loss, and increased caregiver demands (6). Because DLLA represents an advanced manifestation of diabetes-related lower-limb disease, the early identification of modifiable risk factors and reliable predictive markers is essential. Such identification may help prevent progression to severe and irreversible outcomes and support the development of targeted preventive strategies.

In geriatric and chronic disease epidemiology, functional status—commonly assessed through activities of daily living (ADL)—is a critical indicator of overall health reserve and independence among older adults (7). ADL assessments evaluate an individual’s ability to perform basic self-care tasks and reflect the integrated functioning of multiple physiological systems, including musculoskeletal strength, neurological function, cardiovascular integrity, and overall frailty status (8). Extensive evidence has shown that ADL impairment is associated with adverse health outcomes, including increased all-cause mortality, higher hospitalization rates, and elevated risks of cardiovascular events (9, 10). In individuals with diabetes, several mechanisms—such as peripheral neuropathy, peripheral arterial disease, microvascular injury, and diabetes-related sarcopenia—may accelerate lower-limb functional decline and reduce ADL performance (11). These mechanisms support the hypothesis that poorer baseline ADL, and particularly unfavorable ADL trajectories over time, may serve as important predictors of lower-limb complications in individuals with diabetes. Nevertheless, systematic longitudinal studies examining this relationship remain limited.

A major limitation of the existing literature is that most studies rely solely on baseline measurements of functional status. Such an approach fails to capture the dynamic and heterogeneous nature of ADL trajectories among older adults with chronic diseases (12). As individuals age and chronic conditions progress, ADL performance may follow distinct patterns, including stable maintenance, gradual decline, rapid deterioration, or persistently low function. Each trajectory pattern may confer different levels of risk for adverse health outcomes (13). Advances in statistical methodology, particularly group-based trajectory modeling (GBTM), enable the identification of latent subgroups within heterogeneous populations that share similar longitudinal patterns. This approach provides a more nuanced understanding of functional changes over time (14). However, few studies have applied trajectory-based methods to investigate ADL patterns and their association with DLLA. Moreover, most existing research has been conducted within single-country or single-region settings, which may limit generalizability and obscure potential cross-national differences (1). Additional gaps include limited investigation of potential nonlinear relationships between ADL levels and diabetic complications, as well as insufficient exploration of psychological factors such as depression. These factors may exacerbate both functional decline and complication risk among individuals with diabetes (15).

Large, harmonized longitudinal cohort studies provide an ideal framework to address these limitations. The Health and Retirement Study (HRS) in the United States and the English Longitudinal Study of Ageing (ELSA) in the United Kingdom are nationally representative surveys that recruit community-dwelling middle-aged and older adults and conduct repeated standardized assessments over extended follow-up periods (16, 17). Both cohorts collect comprehensive information on health conditions—including diabetes and its complications—functional status through detailed ADL and instrumental ADL assessments, depressive symptoms, socioeconomic characteristics, and lifestyle behaviors. These data allow robust modeling of within-individual changes and enable the investigation of long-term outcomes. Cross-national comparisons between the two cohorts further enhance external validity by evaluating whether associations remain consistent across different healthcare systems and sociocultural contexts.

Therefore, this study aimed to achieve the following objectives (1): to quantify the prospective association between baseline ADL levels and the risk of DLLA using harmonized data from HRS and ELSA (2); to apply group-based trajectory modeling to identify distinct longitudinal patterns of ADL change and examine their differential associations with subsequent DLLA incidence; and (3) to employ complementary analytical strategies—including competing risk models, discrete-time survival regression, and restricted cubic splines—to investigate potential nonlinear relationships while also examining the mediating role of depressive symptoms. Through these analyses, this study seeks to provide new epidemiological insights into the dynamic relationship between functional status trajectories and the risk of lower-limb complications among community-dwelling adults with diabetes, thereby supporting improved early risk stratification and preventive interventions.

## 2 Materials and methods

### 2.1 Study population

The English Longitudinal Study of Ageing (ELSA), conducted by a consortium led by University College London and the National Centre for Social Research, was designed to monitor the health, economic conditions, and social circumstances of adults aged ≥50 years living in private households in England. The Health and Retirement Study (HRS), conducted by the University of Michigan with support from the National Institute on Aging, aims to examine the dynamic interactions among health, economic, and social factors in adults aged ≥51 years within the non-institutionalized civilian population of the United States. The present study was based on a longitudinal cohort analysis using data from HRS and ELSA. Both studies are nationally representative ongoing cohort investigations focusing on community-dwelling older adults. Standardized interviews and physical examinations are conducted biennially to collect detailed information on sociodemographic characteristics, health conditions, functional status, and health-related behaviors.

**Figure 1:**
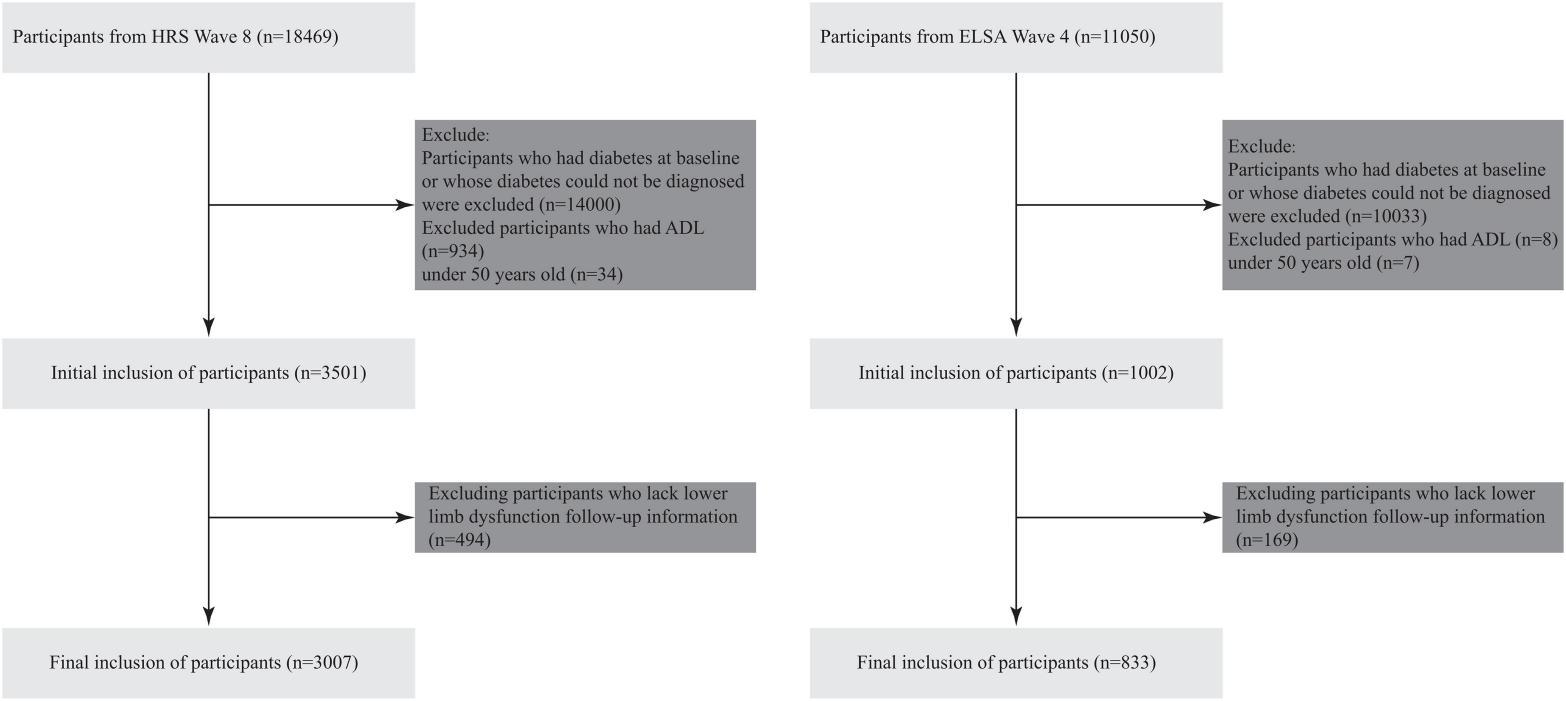
Flowchart of participant selection.

Within the HRS database, 18, 469 participants were initially identified. After applying predefined exclusion criteria, 15, 462 participants were excluded. The exclusions included (1): individuals without diabetes at baseline or without diagnostic information on diabetes (n = 14, 000) (2); participants without ADL assessment data during the first three survey waves (n = 934) (3); individuals younger than 50 years (n = 34); and (4) participants lacking follow-up information on lower-limb functional outcomes (n = 494). After these exclusions, 3, 007 participants from the HRS cohort were included in the final analysis (**Figure 1**).

In the ELSA database, 11, 050 participants were initially identified. A total of 10, 217 participants were excluded according to the eligibility criteria. These exclusions included (1): individuals without diabetes at baseline or without information on diabetes diagnosis (n = 10, 033) (2); participants without ADL data during the first three survey waves (n = 8) (3); individuals younger than 50 years (n = 7); and (4) participants lacking follow-up information on lower-limb functional outcomes (n = 169). Ultimately, 833 participants from the ELSA cohort were included in the present study (**Figure 1**).

### 2.2 Assessment of ADL scores and construction of ADL trajectories

ADL were used to evaluate functional status related to the ability to perform basic self-care tasks. In this study, ADL was assessed using standardized scales available within the cohort databases. The assessment included six fundamental activities: dressing, eating, bathing, toileting, transferring between bed and chair, and walking indoors. For each activity, participants reported whether they experienced difficulty performing the task or required assistance. A score of 1 was assigned if difficulty or assistance was reported; otherwise, a score of 0 was assigned. The scores across all six items were summed to generate a total ADL score ranging from 0 to 6, with higher scores indicating greater functional limitation. To characterize heterogeneity in functional changes over time, longitudinal trajectories of ADL scores were constructed. Consistent with previous trajectory-based studies, ADL data from three consecutive interview waves were used for trajectory modeling in each cohort. Only participants with valid ADL measurements across all three waves were included.

The time variable was defined as the number of years since the baseline interview. Because approximately two years elapsed between survey waves, this variable captured temporal changes in ADL status. Group-Based Trajectory Modeling (GBTM) was applied to identify latent patterns of ADL change within the population. In these models, time was specified as the independent variable and ADL score as the dependent variable. Models with varying numbers of trajectory groups were estimated.

The optimal trajectory model was selected based on multiple criteria, including the Bayesian Information Criterion (BIC), average posterior probability of assignment (AvePP), adequate group membership proportions, and clinical interpretability. Trajectory groups with AvePP values greater than 0.70 were considered to have acceptable classification accuracy. The identified trajectory groups were subsequently treated as categorical variables in the risk analyses to evaluate the association between distinct ADL trajectory patterns and the risk of lower-limb complications among individuals with diabetes.

In the HRS cohort, ADL trajectories were constructed using data collected at Waves 9–11, and follow-up for incident DLLA began at Wave 12. In the ELSA cohort, ADL trajectories were constructed using data collected at Waves 4–6, and follow-up for incident DLLA began at Wave 7. Thus, exposure assessment preceded outcome ascertainment in both cohorts.

### 2.3 Assessment of diabetes-related lower-limb amputation

Because detailed information on diabetic foot ulcers, infection, gangrene, and other diabetes-related lower-limb complications was not consistently available across survey waves in either HRS or ELSA, DLLA was selected as the study outcome. Lower-limb amputation represents a severe and clinically meaningful endpoint of diabetes-related lower-limb disease.

Participants’ diabetes status and lower-limb functional outcomes were assessed using questionnaire-based surveys. Diabetes status was determined based on self-reported responses during the interviews. Individuals were classified as having diabetes if they reported that a physician had diagnosed them with diabetes or if they indicated that they currently had diabetes. After identifying participants with diabetes, lower-limb complications were further assessed. Participants who responded affirmatively to the question regarding whether they had undergone a lower-limb amputation were classified as having DLLA. The available survey items did not distinguish between major and minor amputations; therefore, all reported lower-limb amputations, including toe amputations, were included in the outcome definition. Detailed information regarding outcome ascertainment, coding procedures, survey items, and follow-up assessment across HRS and ELSA is provided in **Supplementary Table 2**.

To ensure the correct temporal relationship between exposure and outcome, all participants included in the trajectory modeling phase (the first three survey waves) had no prior history of DLLA. Individuals with DLLA at baseline were excluded. Follow-up for DLLA began in the survey wave immediately after the trajectory modeling phase and continued until the occurrence of DLLA, loss to follow-up, or the end of follow-up. This design ensured that ADL trajectories preceded the outcome and minimized the potential for reverse causality.

To ensure cross-cohort comparability, harmonized definitions and analytical procedures were applied across HRS and ELSA whenever possible. ADL scores were derived from the same six activities of daily living and calculated using identical scoring methods in both cohorts. Diabetes status was defined using comparable self-reported physician-diagnosed diabetes measures. DLLA was consistently defined as self-reported lower-limb amputation among participants with diabetes. Covariates were harmonized using comparable coding schemes across cohorts. Although the calendar years differed between studies, ADL trajectories were constructed using three consecutive survey waves and follow-up for DLLA began in the subsequent wave in both cohorts. Missing data were handled using the same complete-case approach across cohorts. Detailed harmonization procedures for key variables across HRS and ELSA are presented in **Supplementary Table 3**.

#### 2.3.1 Covariates

Several demographic and lifestyle characteristics were collected and included as covariates in the analyses. These variables included age, sex, race, education, wealth, BMI, drinking status, smoking status, cancer, and physical activity. Smoking status was categorized as non-current smokers and current smokers. Drinking status was classified as current non-drinkers and current drinkers. Body mass index (BMI) was calculated as body weight in kilograms divided by the square of height in meters (kg/m²). Diabetes status was defined based on the survey question “Doctor told you have diabetes”.

#### 2.3.2 Statistical analyses

Continuous variables were summarized using means and standard deviations (SD), whereas categorical variables were presented as counts and percentages. Differences between groups were assessed using the chi-square test or Student’s t-test, as appropriate. To identify distinct patterns of ADL change over time, GBTM based on a finite mixture modeling framework was applied. This method classifies individuals with similar developmental trajectories into latent subgroups using maximum likelihood estimation. Trajectory modeling was performed separately for each cohort.

The models incorporated ADL data from the first three interview waves, and the time variable represented the number of years since baseline. Models with one to three trajectory groups were estimated. The optimal number of trajectory groups was determined by jointly considering the Bayesian Information Criterion (BIC), Akaike Information Criterion (AIC), log-likelihood values, and the mean posterior probability of group membership. The model selection criteria included the lowest BIC value, a minimum group proportion greater than 5%, and an average posterior probability of at least 0.70. Each participant was assigned to a trajectory group based on the maximum posterior probability principle. Multivariable Cox proportional hazards regression models were used to evaluate the associations between ADL scores, ADL trajectories, and the risk of DLLA. Event time was defined as the interval between the end of the three-wave trajectory assessment period and the first occurrence of DLLA, censoring, or the end of follow-up. Hazard ratios (HR) and 95% confidence intervals (CI) were calculated after adjusting for potential confounders. Four models were constructed. Model 1 was an unadjusted model. Model 2 adjusted for age, sex, race, education, wealth, and BMI. Model 3 additionally adjusted for drinking status and smoking status. Model 4 further included cancer and physical activity. Because death may influence the occurrence of DLLA, competing risk regression analyses were performed using the Fine–Gray subdistribution hazard model, with death treated as a competing event. Subdistribution hazard ratios (sHR) and 95% confidence intervals were reported to evaluate the robustness of the findings.

To further assess the stability of the associations under an alternative time structure, discrete-time logistic regression models were also fitted using follow-up waves as the time unit. The dataset was reshaped into a person-period format, in which each participant contributed one observation per follow-up wave until the first occurrence of DLLA or censoring.

Restricted cubic spline (RCS) analyses were conducted to examine potential nonlinear associations between ADL scores and DLLA risk. The inflection point was identified using a data-driven two-piecewise Cox regression model. The threshold value corresponded to the point that provided the best model fit based on the likelihood profile. Subgroup analyses were also performed to evaluate whether the associations between ADL, ADL trajectories, and DLLA risk were consistent across different population groups.

Mediation analyses were conducted to evaluate whether depressive symptoms mediated the association between ADL and DLLA. ADL was specified as the exposure, depressive symptoms as the mediator, and DLLA as the outcome. All mediation models were adjusted for the same covariates included in the fully adjusted Cox models. Total effects, direct effects, indirect effects, proportions mediated, and corresponding 95% confidence intervals were estimated. Because ADL was assessed before outcome ascertainment, the temporal ordering of exposure, mediator, and outcome was preserved. However, the mediation results should be interpreted cautiously because causal mediation assumptions cannot be fully verified in observational studies.

The proportional hazards assumption was evaluated using Schoenfeld residual tests for all Cox proportional hazards models, and no substantial violations were observed. Participants with missing values for exposure variables, outcomes, or key covariates were excluded from the corresponding analyses. Group-based trajectory models were evaluated according to the BIC, AvePP, trajectory group size, and clinical interpretability. Trajectory models with AvePP values greater than 0.70 for all groups were considered acceptable. Detailed model fit indices are provided in **Supplementary Table 1**.

Sampling weights were not applied in the present analyses. The primary objective of this study was to investigate longitudinal associations and trajectory patterns rather than to generate nationally representative population estimates. Therefore, unweighted analyses were performed, consistent with previous trajectory-based longitudinal studies using HRS and ELSA data.

All statistical analyses were conducted using IBM SPSS Statistics (version 24.0) and R software (version 4.3.0). A two-sided p-value < 0.05 was considered statistically significant.

## 3 Results

### 3.1 Baseline characteristics of participants according to DLLA status

**Tables 1** and **2** present the baseline characteristics of participants from the HRS and ELSA cohorts stratified by the occurrence of DLLA. In the HRS cohort, 3, 007 participants without DLLA during the first three survey waves were included in the analysis. The mean age was 68.67 years (SD = 9.14), including 1, 653 females (54.97%) and 1, 354 males (45.03%). During a median follow-up period of approximately two survey waves (approximately four years), 986 participants developed DLLA, with a mean age of 65.09 years (SD = 7.60). Compared with participants who did not develop DLLA, those who experienced DLLA were more likely to be female (63.39% vs 36.61%, p < 0.001) and younger (mean age: 65.09 vs 70.41 years, p<0.001). They also had higher BMI (31.86 vs 30.31, p < 0.001), and a greater prevalence of smoking (55.07% vs 44.93%, p = 0.003). In addition, participants with DLLA had higher CES-D scores (1.96 vs 1.79, p = 0.048) and higher ADL scores (0.71 vs 0.43, p < 0.001) (**Table 1**).

**Table 1:** Baseline characteristics of participants in the HRS cohort stratified by DLLA status.

Characteristic	N	Overall N = 3, 007	No DLLA N = 2, 021	DLLA N = 986	p-value
Sex^b^, n(%)	3, 007				<0.001
FeMale		1, 653 (54.97%)	1, 028 (50.87%)	625 (63.39%)	
Male		1, 354 (45.03%)	993 (49.13%)	361 (36.61%)	
Age(year)a, Mean ± SD	3, 007	68.67 ± 9.14	70.41 ± 9.33	65.09 ± 7.60	<0.001
Race^b^, n(%)	3, 007				0.024
Non-Hispanic white		1, 881 (62.55%)	1, 299 (64.28%)	582 (59.03%)	
Non-Hispanic Blacks		627 (20.85%)	412 (20.39%)	215 (21.81%)	
Hispanic		414 (13.77%)	257 (12.72%)	157 (15.92%)	
Other Race		85 (2.83%)	53 (2.62%)	32 (3.25%)	
Education^b^, n(%)	3, 007				0.004
High school or less		1, 012 (33.63%)	714 (35.35%)	297 (30.12%)	
Some college		1, 527 (50.80%)	985 (48.76%)	542 (54.97%)	
College graduate or above		468 (15.57%)	321 (15.89%)	147 (14.91%)	
Wealth ^a^, Mean ±	3, 007	337, 025.00 ± 883, 047.13	344, 281.85 ± 984, 304.65	322, 150.66 ± 626, 473.76	0.530
BMI ^a^, Mean ±	3, 007	30.82 ± 6.49	30.31 ± 6.61	31.86 ± 6.12	<0.001
Smoking status^b^, n(%)	3, 007				0.003
No		1, 236 (41.10%)	793 (39.24%)	443 (44.93%)	
Yes		1, 771 (58.90%)	1, 228 (60.76%)	543 (55.07%)	
Drinking status^b^, n(%)	3, 007				<0.001
No		1, 917 (63.75%)	1, 331 (65.86%)	586 (59.43%)	
Yes		1, 090 (36.25%)	690 (34.14%)	400 (40.57%)	
Physical activity ^b^, n(%)	3, 007				<0.001
No		1, 193 (39.67%)	857 (42.40%)	336 (34.08%)	
Yes		1, 814 (60.33%)	1, 164 (57.60%)	650 (65.92%)	
Cancer^b^, n(%)	3, 007				<0.001
No		2, 566 (85.33%)	1, 665 (82.38%)	901 (91.38%)	
Yes		441 (14.67%)	356 (17.62%)	85 (8.62%)	
CSE-D^a^, Mean ± SD	3, 007	1.85 ± 2.15	1.79 ± 2.08	1.96 ± 2.28	0.048
ADL Score^a^, Mean ± SD	3, 007	0.62 ± 1.29	0.43 ± 1.00	0.71 ± 1.40	<0.001
ADL trajectory^b^, n(%)	3, 007				<0.001
Stable low		2, 147 (71.40%)	1, 349 (66.75%)	798 (80.93%)	
Stable rise		700 (23.28%)	534 (26.42%)	166 (16.84%)	
Stable High		160 (5.32%)	138 (6.83%)	22 (2.23%)	

^a^ Student t-test.

^b^ Chi-square test.

SD, standard deviation.

**Table 2:** Baseline characteristics of participants in the ELSA cohort stratified by DLLA status.

Characteristic	N	Overall N = 833	No DLLA N = 579	DLLA N = 254	p-value
Sex^b^, n(%)	833				<0.001
FeMale		379 (45.50%)	241 (41.62%)	138 (54.33%)	
Male		454 (54.50%)	338 (58.38%)	116 (45.67%)	
Age(year)^a^, Mean ± SD	833	67.96 ± 9.44	68.80 ± 10.00	66.04 ± 7.71	<0.001
Race^b^, n(%)	833				0.110
No white		52 (6.24%)	31 (5.35%)	21 (8.27%)	
White		781 (93.76%)	548 (94.65%)	233 (91.73%)	
Education^b^, n(%)	833				0.694
Before high school		442 (53.06%)	315 (54.40%)	127 (50.00%)	
High school		142 (17.05%)	97 (16.75%)	45 (17.72%)	
Junior college		160 (19.21%)	107 (18.48%)	53 (20.87%)	
College graduate or above		89 (10.68%)	60 (10.36%)	29 (11.42%)	
Wealth ^a^, Mean ±	833	231, 942.57 ± 513, 506.78	242, 565.88 ± 593, 702.94	207, 726.44 ± 247, 036.64	0.422
BMI ^a^, Mean ±	833	31.04 ± 5.61	30.23 ± 5.18	32.90 ± 6.10	<0.001
Smoking status^a^, n(%)	833				0.385
No		287 (34.45%)	194 (33.51%)	93 (36.61%)	
Yes		546 (65.55%)	385 (66.49%)	161 (63.39%)	
Drinking status^a^, n(%)	833				0.402
No		131 (15.73%)	87 (15.03%)	44 (17.32%)	
Yes		702 (84.27%)	492 (84.97%)	210 (82.68%)	
Physical activity ^a^, n(%)	833				0.890
No		325 (39.02%)	225 (38.86%)	100 (39.37%)	
Yes		508 (60.98%)	354 (61.14%)	154 (60.63%)	
Cancerb, n(%)	833				0.914
No		743 (89.20%)	516 (89.12%)	227 (89.37%)	
Yes		90 (10.80%)	63 (10.88%)	27 (10.63%)	
CSE-D^a^, Mean ± SD	833	1.79 ± 2.10	1.69 ± 2.02	2.01 ± 2.25	0.054
ADL^a^, Mean ± SD	833	0.60 ± 1.18	0.55 ± 1.16	0.73 ± 1.22	0.048
ADL trajectory ^a^, n(%)	833				0.036
Stable-low		612 (73.47%)	438 (75.65%)	174 (68.50%)	
Stable rise		136 (16.33%)	85 (14.68%)	51 (20.08%)	
Stable-High		85 (10.20%)	56 (9.67%)	29 (11.42%)	

^a^ Student t-test.

^b^ Chi-square test

SD, standard deviation.

In the ELSA cohort, 833 participants without DLLA during the first three survey waves were included. The mean age was 67.96 years (SD = 9.44), and the sample consisted of 379 females (45.50%) and 454 males (54.50%). During a median follow-up period of approximately two survey waves (approximately four years), 254 participants developed DLLA, with a mean age of 66.04 years (SD = 7.71). Compared with participants who did not develop DLLA, those who developed DLLA were younger (66.04 vs 68.80 years, p < 0.001), had higher BMI values (32.90 vs 30.23, p < 0.001). Furthermore, participants with DLLA had higher CES-D scores (2.01 vs 1.69, p = 0.054) and higher ADL scores (0.73 vs 0.55, p = 0.048) (**Table 2**).

### 3.2 Association between ADL and ADL trajectories with the risk of DLLA

**Table 3** summarizes the associations between baseline ADL, ADL trajectories, and the risk of DLLA in the two cohorts based on Cox proportional hazards regression models. In the crude model, baseline ADL was significantly associated with an increased risk of DLLA (HRS: HR = 1.17, 95% CI: 1.13–1.20, p <.001; ELSA: HR = 1.30, 95% CI: 1.22–1.38, p <.001). After adjustment for potential confounders in Model 4—including age, sex, race, education, wealth, BMI, drinking status, smoking status, physical activity, and cancer—the association remained stable and statistically significant in both cohorts (HRS: adjusted HR = 1.10, 95% CI: 1.07–1.14, p <.001; ELSA: adjusted HR = 1.20, 95% CI: 1.12–1.28, p <.001). These findings indicate that each one-unit increase in ADL score was associated with an approximately 10–20% higher risk of DLLA.

**Table 3:** Associations between ADL and its trajectories and the risk of DLLA.

Exposure Variable	Model 1		Model 2		Model 3		Model 4	
	HR (95%CI)	P-value	HR (95% CI)	P-value	HR (95% CI)	P-value	HR (95% CI)	P-value
HRS								
ADL Score	1.17 (1.13~ 1.20)	<.001***	1.11 (1.08 ~ 1.15)	<.001***	1.12 (1.08 ~ 1.15)	<.001***	1.10 (1.07 ~ 1.14)	<.001***
ADL trajectory								
Stable-low	1.00 (Reference)	–	1.00 (Reference)	–	1.00 (Reference)	–	1.00 (Reference)	–
Stable rise	1.73 (1.58 ~ 1.90)	<.001***	1.55 (1.41 ~ 1.71)	<.001***	1.55 (1.41 ~ 1.71)	<.001***	1.51 (1.37 ~ 1.67)	<.001***
Stable-High	1.71 (1.44 ~ 2.03)	<.001***	1.43 (1.20 ~ 1.71)	<.001***	1.47 (1.23 ~ 1.76)	<.001***	1.36 (1.13 ~ 1.64)	<.001***
P for trend	1.14 (1.11 ~ 1.18)	<.001***	1.09 (1.06 ~ 1.13)	<.001***	1.10 (1.06 ~ 1.13)	<.001***	1.08 (1.04 ~ 1.11)	<.001***
ELSA								
ADL Score	1.30 (1.22 ~ 1.38)	<.001***	1.23 (1.16 ~ 1.32)	<.001***	1.23 (1.15 ~ 1.32)	<.001***	1.20 (1.12 ~ 1.28)	<.001***
ADL trajectory								
Stable-low	1.00 (Reference)	–	1.00 (Reference)	–	1.00 (Reference)	–	1.00 (Reference)	–
Stable rise	2.29 (1.82 ~ 2.87)	<.001***	1.93 (1.53 ~ 2.45)	<.001***	1.94 (1.53 ~ 2.48)	<.001***	1.84 (1.44 ~ 2.36)	<.001***
Stable-High	3.07 (2.35 ~ 3.99)	<.001***	2.51 (1.90 ~ 3.32)	<.001***	2.51 (1.89 ~ 3.32)	<.001***	2.31 (1.72 ~ 3.12)	<.001***
P for trend	1.48 (1.37 ~ 1.61)	<.001***	1.37 (1.26 ~ 1.50)	<.001***	1.37 (1.25 ~ 1.50)	<.001***	1.33 (1.21 ~ 1.46)	<.001***

Model 1: Crude.

Model 2: Adjust: Age, Sex, Race, Education, marital status, Wealth, BMI.

Model 3: Adjust: Age, Sex, Race, Education, marital status, Wealth, BMI, Drinking status, Smoking status.

Model 4: Adjust: Age, Sex, Race, Education, marital status, Wealth, BMI, Drinking status, Smoking status, Physical activity, Cancer.

Significance: * P < 0.05, ** P< 0.01, *** P < 0.001.

**Figure 2:**
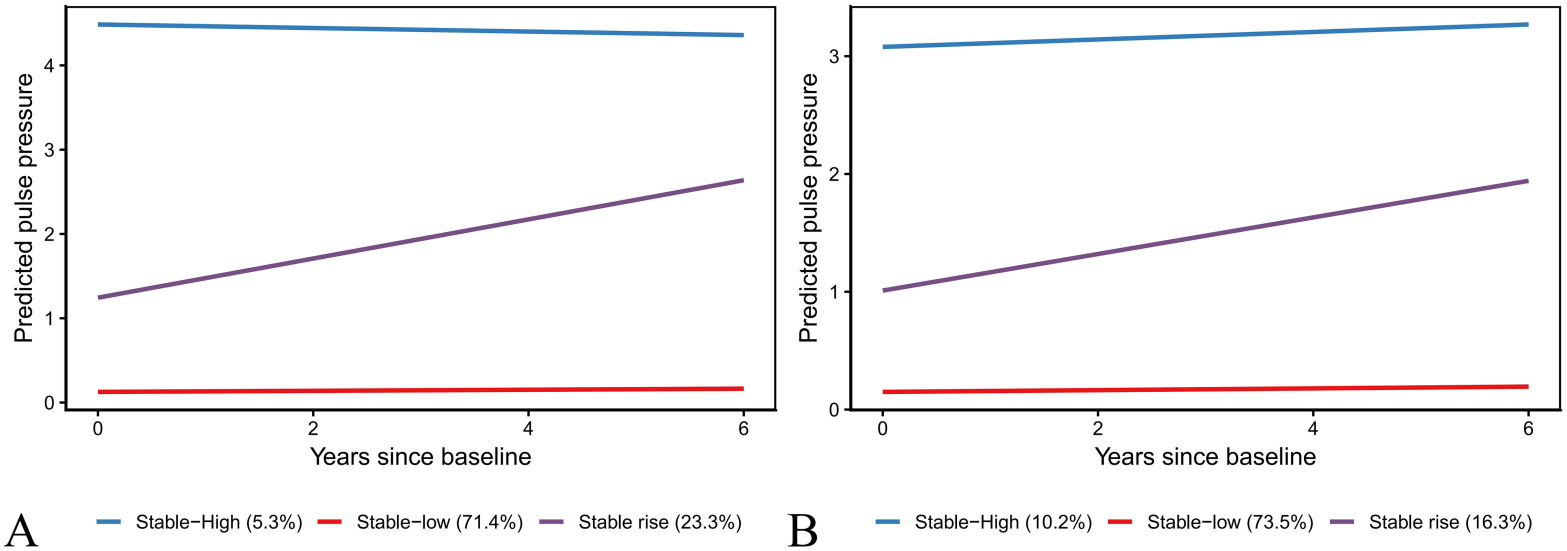
ADL trajectories identified in the study population using trajectory analysis: **(A)** HRS cohort; **(B)** ELSA cohort.

GBTM was used to identify longitudinal patterns of ADL change. Based on the BIC, three trajectory groups were identified: Stable-low, Stable-rise, and Stable-high (**Figure 2**). The numbers of DLLA events observed within each trajectory group and detailed model evaluation metrics are presented in **Supplementary Table 1**. Using the Stable-low group as the reference, both the Stable-rise and Stable-high groups exhibited significantly higher risks of DLLA after adjustment for potential confounders.

Across the two cohorts, participants in the Stable-rise group showed a 51–84% higher risk of DLLA (HRS: adjusted HR = 1.51, 95% CI: 1.37–1.67, p <.001; ELSA: adjusted HR = 1.84, 95% CI: 1.44–2.36, p <.001). Similarly, the Stable-high group demonstrated an increased risk ranging from 36% to more than twofold (HRS: adjusted HR = 1.36, 95% CI: 1.13–1.64, p <.001; ELSA: adjusted HR = 2.31, 95% CI: 1.72–3.12, p <.001) (**Table 4**). Trend analyses further indicated that the risk of DLLA increased progressively with worsening ADL trajectory patterns (HRS: adjusted HR = 1.08, 95% CI: 1.04–1.11, p <.001; ELSA: adjusted HR = 1.33, 95% CI: 1.21–1.46, p <.001).

**Table 4:** Fine–Gray regression analysis examining the associations between ADL, its trajectories, and the risk of DLLA.

Exposure variable	Model 1		Model 2		Model 3		Model 4	
	sHR (95% CI)	P-value	sHR (95% CI)	P-value	sHR (95% CI)	P-value	sHR (95% CI)	P-value
HRS								
ADLScore	1.09 (1.08 ~ 1.10)	<.001***	1.06 (1.04 ~ 1.07)	<.001***	1.06 (1.04 ~ 1.07)	<.001***	1.05 (1.04 ~ 1.07)	<.001***
ADLtrajectory								
Stable-low	1.00 (Reference)	–	1.00 (Reference)	–	1.00 (Reference)	–	1.00 (Reference)	–
Stable rise	1.37 (1.31 ~ 1.44)	<.001***	1.28 (1.22 ~ 1.34)	<.001***	1.28 (1.22 ~ 1.34)	<.001***	1.26 (1.20 ~ 1.33)	<.001***
StableHigh	1.36 (1.26 ~ 1.47)	<.001***	1.21 (1.12 ~ 1.31)	<.001***	1.22 (1.13 ~ 1.32)	<.001***	1.19 (1.09 ~ 1.29)	<.001***
P for trend	1.08 (1.07 ~ 1.10)	<.001***	1.05 (1.03 ~ 1.07)	<.001***	1.05 (1.04 ~ 1.07)	<.001***	1.04 (1.03 ~ 1.06)	<.001***
ELSA								
ADLScore	1.20 (1.15 ~ 1.25)	<.001***	1.16 (1.11 ~ 1.21)	<.001***	1.16 (1.11 ~ 1.21)	<.001***	1.13 (1.08 ~ 1.19)	<.001***
ADL trajectory								
Stable-low	1.00 (Reference)	–	1.00 (Reference)	–	1.00 (Reference)	–	1.00 (Reference)	–
Stable rise	1.84 (1.58 ~ 2.14)	<.001***	1.62 (1.38 ~ 1.91)	<.001***	1.62 (1.38 ~ 1.91)	<.001***	1.57 (1.32 ~ 1.85)	<.001***
Stable-High	2.15 (1.86 ~ 2.50)	<.001***	1.85 (1.57 ~ 2.17)	<.001***	1.84 (1.57 ~ 2.17)	<.001***	1.75 (1.46 ~ 2.11)	<.001***
P for trend	1.31 (1.25 ~ 1.38)	<.001***	1.24 (1.18 ~ 1.30)	<.001***	1.24 (1.17 ~ 1.30)	<.001***	1.21 (1.14 ~ 1.29)	<.001***

Model 1: Crude.

Model 2: Adjust: Age, Sex, Race, Education, marital status, Wealth, BMI.

Model 3: Adjust: Age, Sex, Race, Education, marital status, Wealth, BMI, Drinking status, Smoking status.

Model 4: Adjust: Age, Sex, Race, Education, marital status, Wealth, BMI, Drinking status, Smoking status, Physical activity, Cancer.

Significance: * P < 0.05, ** P < 0.01, *** P < 0.001.

**Figure 3:**
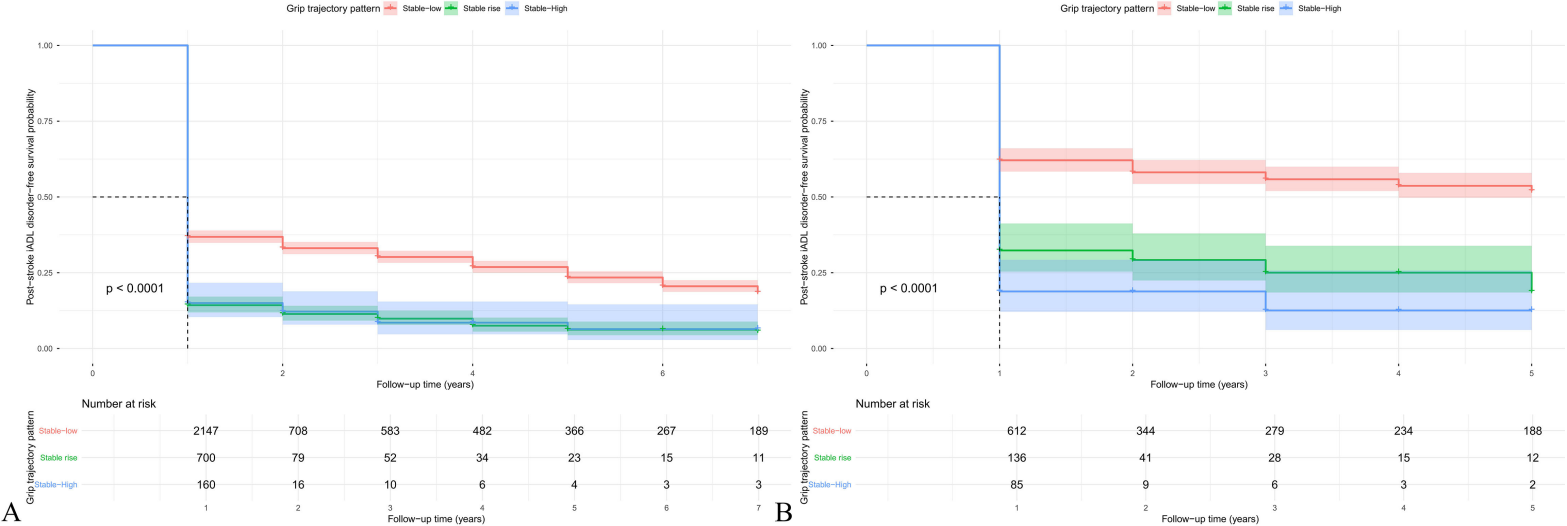
Kaplan–Meier curves illustrating differences in DLLA-free survival among participants with different ADL trajectory groups: **(A)** HRS cohort; **(B)** ELSA cohort.

Kaplan–Meier survival analyses demonstrated significant differences in DLLA-free survival probabilities across the ADL trajectory groups in both cohorts. Participants in the Stable-low group exhibited significantly higher survival probabilities than those in the Stable-rise and Stable-high groups (HRS: log-rank p < 0.0001; ELSA: log-rank p < 0.0001) (**Figure 3**).

### 3.3 Competing risk analysis considering death

Fine–Gray competing risk regression analyses were conducted to evaluate the associations between ADL, ADL trajectories, and DLLA while accounting for death as a competing event (**Table 4**). The results were consistent with those obtained from the Cox proportional hazards models. After adjustment for confounders, ADL remained independently associated with the subdistribution risk of DLLA (HRS: adjusted sHR = 1.05, 95% CI: 1.04–1.07, p <.001; ELSA: adjusted sHR = 1.13, 95% CI: 1.08–1.19, p <.001).

Compared with participants in the Stable-low trajectory group, those in the Stable-rise and Stable-high groups continued to exhibit significantly higher risks of DLLA. The Stable-rise group showed a 26–57% increased risk (HRS: adjusted sHR = 1.26, 95% CI: 1.20–1.33, p <.001; ELSA: adjusted sHR = 1.57, 95% CI: 1.32–1.85, p <.001). The Stable-high group demonstrated a 19–75% increased risk (HRS: adjusted sHR = 1.19, 95% CI: 1.09–1.29, p <.001; ELSA: adjusted sHR = 1.75, 95% CI: 1.46–2.11, p <.001). Trend analyses based on the Fine–Gray models were also statistically significant (HRS: adjusted sHR = 1.04, 95% CI: 1.03–1.06, p <.001; ELSA: adjusted sHR = 1.21, 95% CI: 1.14–1.29, p <.001). The magnitude and direction of these associations were comparable to those observed in the Cox models, suggesting that the relationship between ADL trajectories and DLLA risk was not substantially affected by the competing risk of death.

### 3.4 Discrete-time logistic regression analysis

Discrete-time logistic regression models were constructed using follow-up waves as the time unit. The dataset was transformed into a person-period format, allowing each participant to contribute one observation per follow-up wave until the occurrence of DLLA or censoring. ADL and its trajectories were significantly associated with the risk of DLLA within each follow-up interval.

In the fully adjusted model, ADL remained positively associated with DLLA risk in both cohorts (HRS: adjusted OR = 1.32, 95% CI: 1.23–1.43, p <.001; ELSA: adjusted OR = 1.47, 95% CI: 1.28–1.69, p <.001). Compared with the Stable-low group, participants in the Stable-rise and Stable-high groups had significantly higher risks of DLLA. The Stable-rise group showed a 1.36–1.41-fold higher risk (HRS: adjusted OR = 2.41, 95% CI: 1.98–2.95, p <.001; ELSA: adjusted OR = 2.36, 95% CI: 1.64–3.42, p <.001), while the Stable-high group exhibited a 1.24–3.29-fold higher risk (HRS: adjusted OR = 2.24, 95% CI: 1.49–3.41, p <.001; ELSA: adjusted OR = 4.29, 95% CI: 2.53–7.43, p <.001).

The direction and magnitude of the associations observed in the discrete-time models were consistent with those obtained from the Cox and competing risk analyses, indicating the robustness of the findings across different time-modeling strategies (**Table 5**).

**Table 5:** Discrete-time logistic regression analysis examining the associations between ADL, its trajectories, and the risk of DLLA.

Exposure variable	Model 1		Model 2		Model 3		Model 4	
	OR(95% CI)	P-value	OR (95% CI)	P-value	OR (95% CI)	P-value	OR (95% CI)	P-value
HRS								
ADL Score	1.45 (1.35 ~ 1.56)	<0.001***	1.34 (1.25 ~ 1.44)	<0.001***	1.35 (1.26 ~ 1.46)	<0.001***	1.32 (1.23 ~ 1.43)	<0.001***
ADLtrajectory								
Stable-low	1.00 (Reference)	–	1.00 (Reference)	–	1.00 (Reference)	–	1.00 (Reference)	–
Stable rise	2.88 (2.39 ~ 3.47)	<0.001***	2.46 (2.03 ~ 3.00)	<0.001***	2.51 (2.07 ~ 3.07)	<0.001***	2.41 (1.98 ~ 2.95)	<0.001***
Stable-High	2.80 (1.96 ~ 4.08)	<0.001***	2.25 (1.54 ~ 3.35)	<0.001***	2.47 (1.66 ~ 3.74)	<0.001***	2.24 (1.49 ~ 3.41)	<0.001***
P for trend	1.38 (1.28 ~ 1.50)	<0.001***	1.29 (1.19 ~ 1.40)	<0.001***	1.33 (1.22 ~ 1.45)	<0.001***	1.29 (1.19 ~ 1.41)	<0.001***
ELSA								
ADL Score	1.66 (1.46 ~ 1.90)	<0.001***	1.51 (1.33 ~ 1.73)	<0.001***	1.51 (1.33 ~ 1.74)	<0.001***	1.47 (1.28 ~ 1.69)	<0.001***
ADLtrajectory								
Stable-low	1.00 (Reference)	–	1.00 (Reference)	–	1.00 (Reference)	–	1.00 (Reference)	–
Stable rise	3.10 (2.21 ~ 4.37)	<0.001***	2.44 (1.72 ~ 3.49)	<0.001***	2.47 (1.73 ~ 3.55)	<0.001***	2.36 (1.64 ~ 3.42)	<0.001***
Stable-High	6.08 (3.73 ~ 10.17)	<0.001***	4.57 (2.76 ~ 7.74)	<0.001***	4.58 (2.77 ~ 7.76)	<0.001***	4.29 (2.53 ~ 7.43)	<0.001***
P for trend	2.00 (1.70 ~ 2.36)	<0.001***	1.76 (1.49 ~ 2.09)	<0.001***	1.76 (1.49 ~ 2.09)	<0.001***	1.71 (1.44 ~ 2.05)	<0.001***

Model 1: Crude.

Model 2: Adjust: Age, Sex, Race, Education, marital status, Wealth, BMI.

Model 3: Adjust: Age, Sex, Race, Education, marital status, Wealth, BMI, Drinking status, Smoking status.

Model 4: Adjust: Age, Sex, Race, Education, marital status, Wealth, BMI, Drinking status, Smoking status, Physical activity, Cancer.

Significance: * P < 0.05, ** P< 0.01, *** P < 0.001.

### 3.5 Subgroup analyses

**Figure 4:**
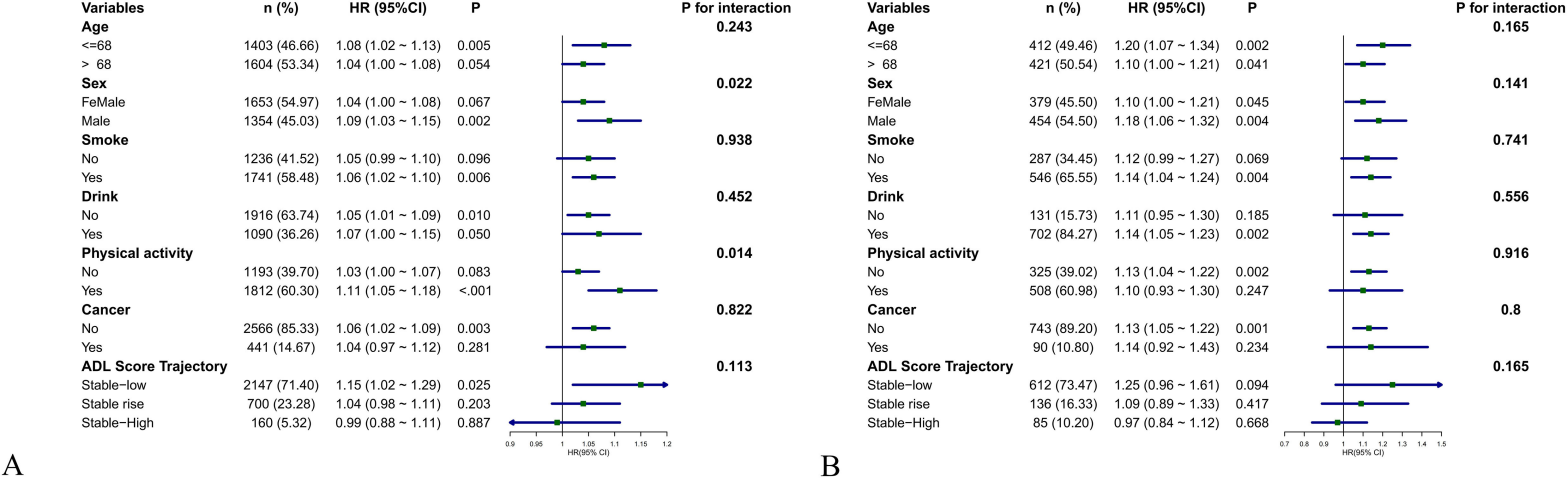
Forest plots showing the associations between ADL scores and the risk of DLLA across different subgroups: **(A)** HRS cohort; **(B)** ELSA cohort.

Subgroup analyses were performed to determine whether the association between ADL and DLLA risk differed across population subgroups. Stratified analyses were conducted according to sex, age, physical activity, and chronic conditions. In the HRS cohort, the association between ADL and DLLA risk was stronger among participants younger than 68 years, males, and smokers. Similar patterns were observed in the ELSA cohort, where stronger associations were also evident among individuals younger than 68 years, males, and smokers. Interaction tests indicated significant interactions between ADL and sex (P for interaction = 0.022) and between ADL and physical activity (P for interaction = 0.014) in the HRS cohort. No significant interactions were detected for other subgroup variables (P for interaction > 0.05). In the ELSA cohort, no statistically significant interactions were observed across the examined subgroups (**Figure 4**).

### 3.6 Linear and nonlinear associations between ADL and DLLA risk

**Figure 5:**
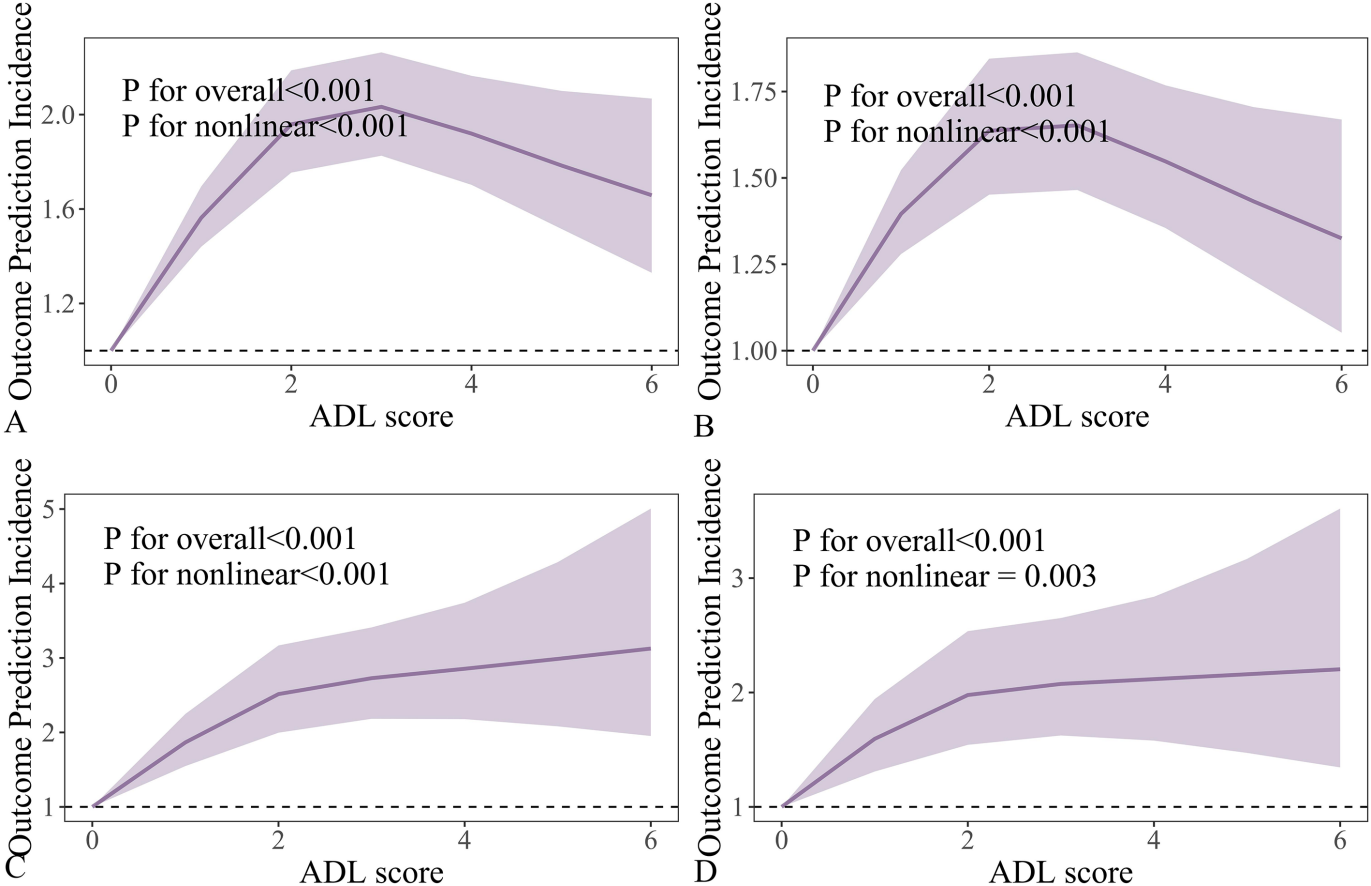
Restricted cubic spline analyses illustrating the linear and nonlinear associations between ADL and the risk of DLLA: **(A)** unadjusted model in the HRS cohort; **(B)** fully adjusted model in the HRS cohort; **(C)** unadjusted model in the ELSA cohort; **(D)** fully adjusted model in the ELSA cohort.

RCS analyses were conducted to examine the potential nonlinear relationship between ADL scores and the risk of DLLA. The results are presented in **Figure 5**. Before adjustment for confounders, significant nonlinear associations were observed in both cohorts (HRS: p for nonlinear <.001 and p for overall <.001; ELSA: p for nonlinear <.001 and p for overall <.001). After adjusting for potential confounders, the nonlinear relationship remained significant in both cohorts (HRS: p for nonlinear < 0.001 and p for overall < 0.001; ELSA: p for nonlinear = 0.003 and p for overall < 0.001). Threshold effect analyses identified an inflection point at an ADL score of 2 in both cohorts using a data-driven two-piecewise Cox regression approach. In the HRS cohort, ADL scores below 2 were significantly associated with increased DLLA risk (adjusted HR = 1.09, 95% CI: 1.07–1.14, p <.001). However, when ADL scores exceeded 2, the association was no longer statistically significant (adjusted HR = 0.97, 95% CI: 0.84–1.17, p = 0.514) (**Table 6**). A similar pattern was observed in the ELSA cohort. ADL scores below 2 were significantly associated with a higher risk of DLLA (adjusted HR = 1.46, 95% CI: 1.27–1.69, p <.001), whereas the association was not significant when ADL scores exceeded 2 (adjusted HR = 0.99, 95% CI: 0.85–1.15, p = 0.898) (**Table 7**). Although the associations above the threshold were not statistically significant, the confidence intervals became wider at higher ADL values, which may partly reflect the relatively small number of participants with severe functional impairment.

**Table 6:** Threshold effect analysis investigating the nonlinear association between ADL scores and the risk of DLLA in the HRS cohort.

Parameter	HR (95% CI), P-value
Model 1 Fitting model by standard linear regression	1.10 (1.07 ~ 1.14), <.001
Model 2 Fitting model by two-piecewise linear regression	
Inflection point	2
<2	1.09(1.07-1.14), <.001
>2	0.97(0.84-1.17), 0.514
P for likelihood ratio test	0.045

Model 1: Standard linear regression with ADL as a continuous predictor.

Model 2: Two-piecewise linear regression with ADL split at the inflection point.

Significance: P < 0.05 indicates statistical significance.

**Table 7:** Threshold effect analysis investigating the nonlinear association between ADL scores and the risk of DLLA in the ELSA cohort.

Parameter	HR (95% CI), P-value
Model 1 Fitting model by standard linear regression	1.2(1.12-1.28), <.001
Model 2 Fitting model by two-piecewise linear regression	
Inflection point	2
<2	1.46(1.27-1.69), <.001
>2	0.99(0.85-1.15), 0.898
P for likelihood ratio test	0.003

Model 1: Standard linear regression with ADL as a continuous predictor.

Model 2: Two-piecewise linear regression with ADL split at the inflection point.

Significance: P < 0.05 indicates statistical significance.

### 3.7 Mediation analysis

**Figure 6:**
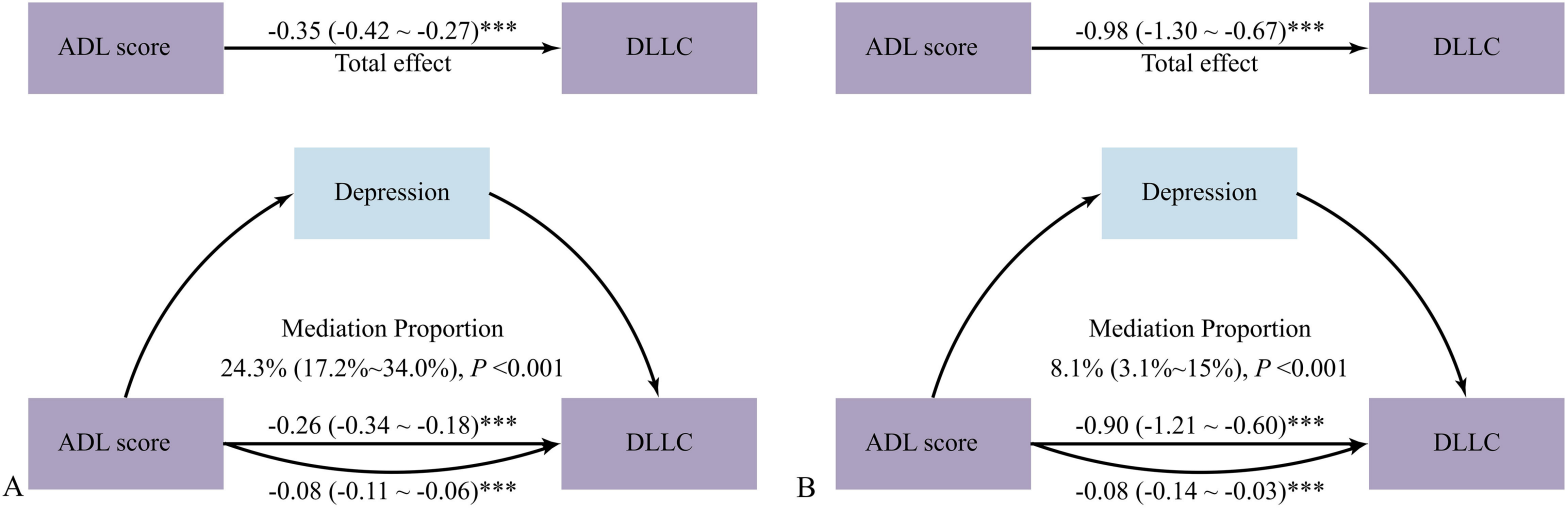
Mediation analysis demonstrating the mediating role of depression in the association between ADL and the risk of DLLA: **(A)** HRS cohort; **(B)** ELSA cohort.

**Figure 6** illustrates the mediating role of depression in the association between ADL and DLLA risk. Depression partially mediated this relationship in both cohorts. Detailed estimates of the total effect, direct effect, indirect effect, proportion mediated, and corresponding 95% confidence intervals are presented in **Figure 6**. In the HRS cohort, the mediation effect accounted for approximately 24.3% of the total effect, whereas in the ELSA cohort it accounted for approximately 8.1%. Both the direct and total effects remained statistically significant in both populations. These findings suggest that depression may partially explain the association between ADL and DLLA; however, the mediation estimates should be interpreted cautiously because the analysis was conducted within an observational framework and cannot establish causality.

## 4 Discussion

This study systematically examined the association between ADL and their longitudinal trajectories with the risk of DLLA using two nationally representative longitudinal cohorts, the HRS and ELSA. The findings demonstrate that higher ADL scores were associated with an increased risk of DLLA, and this association was consistently observed across both independent cohorts. Trajectory analyses further revealed substantial differences in DLLA risk across populations with distinct patterns of functional change. Individuals with persistently poor or progressively deteriorating functional status exhibited a markedly higher risk of DLLA than those with stable and favorable functional status. The robustness of these findings was supported by consistent results obtained from competing risk models and discrete-time models. Restricted cubic spline analyses additionally revealed a nonlinear relationship between ADL and DLLA risk, with a threshold effect observed at an ADL score of approximately 2. Mediation analyses indicated that depressive symptoms partially mediated the association between ADL and DLLA. Collectively, these findings suggest that functional status and its long-term trajectories may serve as important indicators for predicting the risk of DLLA among individuals with diabetes.

The relationship between limitations in activities of daily living and an increased risk of DLLA may be explained by several interacting physiological and behavioral mechanisms. In individuals with diabetes, peripheral neuropathy and peripheral vascular disease commonly impair lower-limb sensation, muscle strength, and blood circulation. These impairments compromise gait stability and reduce the ability to perform basic self-care activities. As ADL limitations worsen, reduced mobility and weight-bearing capacity may further promote lower-limb muscle atrophy, joint stiffness, and microvascular damage. This process may create a detrimental cycle that increases the likelihood of foot trauma, ulceration, and infection (18–20). From a behavioral perspective, worsening functional impairment is often accompanied by reduced physical activity and diminished self-management capacity. Such changes may include inadequate foot examinations, poorer glycemic control, and delayed recognition of early lesions, thereby increasing the risk of complications (21–23). Moreover, ADL limitations are frequently considered markers of broader frailty (24). Frailty is characterized by reduced physiological reserves across multiple organ systems and has been associated with adverse outcomes in individuals with chronic diseases, including accelerated progression of diabetes-related lower-limb disorders (25). ADL impairment may not function solely as an independent risk factor for DLLA but may also represent a clinical marker of broader pathological processes, including frailty, peripheral neuropathy, peripheral vascular disease, sarcopenia, depression, and pre-existing disability. Therefore, the observed associations should be interpreted within the context of overall disease burden and physiological vulnerability. From a clinical perspective, ADL assessment may serve as a practical risk-stratification tool for identifying individuals who may benefit from closer monitoring and comprehensive preventive care.

Trajectory-based analyses represent an important methodological advancement compared with single baseline assessments of functional status. Unlike baseline measurements, group-based trajectory models capture the dynamic and long-term evolution of ADL performance. The present findings indicate that individuals with persistently poor or progressively declining ADL trajectories experienced a substantially higher incidence of DLLA compared with those whose functional status remained stable and favorable. This pattern may reflect the cumulative physiological burden that develops over time. Persistent limitations in physical activity may exacerbate neuropathic and vascular damage, ultimately leading to clinically significant complications (26, 27). Identification of functional trajectories also has important clinical implications. It may facilitate the early identification of individuals at elevated risk of DLLA and support more comprehensive clinical evaluation and risk management (28). However, the present findings should not be interpreted as evidence that improving ADL alone directly reduces the risk of DLLA. Rather, ADL trajectories may provide useful information for risk stratification and clinical decision-making.

The restricted cubic spline and threshold effect analyses revealed a nonlinear dose–response relationship between ADL limitations and DLLA risk. The risk increased most rapidly within the range of mild-to-moderate functional impairment, corresponding to values below the identified threshold. Beyond the identified threshold, the association appeared to attenuate; however, this finding should be interpreted cautiously. The number of participants with severe functional impairment was relatively limited, which may have reduced statistical precision and contributed to wider confidence intervals at higher ADL levels. Therefore, the present findings do not necessarily indicate that risk plateaus beyond an ADL score of 2, and additional studies are needed to confirm the observed threshold effect. From both clinical and public health perspectives, these findings emphasize the importance of early intervention during the initial stages of functional decline. These findings suggest that mild-to-moderate functional impairment may represent an important stage for risk identification and clinical monitoring among individuals with diabetes (27, 29–31).

Depressive symptoms were found to partially mediate the association between ADL and DLLA in both cohorts. This observation aligns with the well-established bidirectional relationship among mental health, functional capacity, and physical complications in individuals with diabetes (32, 33). Depression may influence this pathway through behavioral mechanisms, including reduced motivation for physical activity and poorer adherence to self-care practices (32, 34). In addition, physiological mechanisms may contribute through neuroendocrine dysregulation and systemic inflammatory responses, which can accelerate vascular and neural damage (33, 35). These findings highlight the importance of integrated care approaches that address both physical function and psychological health. For example, routine screening for depressive symptoms combined with multidisciplinary management strategies may help reduce the burden of lower-limb complications (36). Although depression partially mediated the association between ADL and DLLA, this finding should be interpreted cautiously. Depression may represent only one component of a broader network of biological, behavioral, and psychosocial mechanisms linking functional impairment to adverse lower-limb outcomes. Other factors, including frailty, diabetes severity, peripheral neuropathy, vascular disease, social isolation, and disability-related processes, may also contribute to this relationship. Therefore, the mediation results should be viewed as hypothesis-generating rather than evidence of a definitive causal pathway.

Several methodological strengths enhance the credibility and generalizability of the present study. First, the analysis utilized two large, nationally representative longitudinal cohorts with extended follow-up periods, standardized measurements of ADL and outcomes, and comprehensive covariate data. These features provided substantial statistical power and enabled cross-national validation of the findings (16, 17). Second, group-based trajectory modeling allowed a detailed characterization of functional heterogeneity within the population (37). Third, multiple complementary analytical approaches—including Cox proportional hazards models, competing risk regression, discrete-time survival analysis, restricted cubic spline modeling, and mediation analysis—were applied to strengthen the robustness of the results and to account for different sources of bias in longitudinal observational studies (38, 39). Finally, the investigation of nonlinear relationships and mediation pathways provided deeper insights into the mechanisms underlying the observed associations beyond simple correlation analyses.

Despite these strengths, several limitations should be considered. First, the study relied on self-reported measures of ADL and lower-limb complications, with DLLA primarily defined as amputation events. This approach may introduce reporting or recall bias and may underestimate the broader spectrum of DLLA, such as non-amputation ulcers or infections (40). Second, although extensive adjustments were made for demographic characteristics, lifestyle factors, and major comorbidities, several diabetes-specific clinical factors were unavailable or inconsistently measured across the HRS and ELSA cohorts. These factors include diabetes duration, glycemic control (e.g., HbA1c), insulin use, peripheral neuropathy, peripheral arterial disease, prior foot ulceration, foot deformity, medication adherence, and other indicators of diabetes severity. Because these variables are established risk factors for lower-limb complications, residual confounding cannot be completely excluded. Consequently, the observed associations should be interpreted cautiously, and future studies incorporating more detailed diabetes-specific clinical information are needed to validate the present findings. In addition, ADL trajectories were constructed using three repeated assessments. Although this approach is consistent with previous trajectory-based studies and demonstrated acceptable classification performance, the limited number of measurement waves may have reduced the ability to identify more complex functional patterns and may have affected trajectory stability. Third, the study population consisted mainly of middle-aged and older adults from high-income Western countries. Consequently, the generalizability of the findings to younger populations, diverse ethnic groups, or individuals living in low- and middle-income countries may be limited, particularly in settings with different patterns of diabetes epidemiology and healthcare access. Furthermore, although ADL trajectories were established before the start of outcome follow-up, reverse causality cannot be completely excluded. Participants with subclinical lower-limb disease or early pathological changes may already have experienced functional impairment before the occurrence or reporting of DLLA. Therefore, impaired ADL may partially reflect underlying disease progression rather than functioning exclusively as an independent risk factor. Future studies with more detailed clinical assessments and repeated evaluations of lower-limb health are warranted to further clarify the temporal relationship.

## 5 Conclusion

In conclusion, analyses of two large longitudinal cohorts demonstrated significant associations between ADL levels, their long-term trajectories, and the risk of DLLA. Greater functional limitations were associated with an elevated risk of DLLA, and individuals with persistently poor or progressively deteriorating functional trajectories exhibited the highest risk. A nonlinear relationship between ADL and DLLA was also observed, with depressive symptoms partially mediating this association. These findings suggest that routine assessment of functional status may help identify individuals at elevated risk of DLLA. ADL should be considered primarily as a risk-stratification indicator reflecting overall health vulnerability rather than as a direct causal determinant of DLLA.

## Data Availability

The datasets presented in this study can be found in online repositories. The names of the repository/repositories and accession number(s) can be found below: HRS: https://hrsdata.isr.umich.edu/; https://www.elsa-project.ac.uk/.
