# Supplementary material for: Associations of activities of daily living and their trajectories with the risk of diabetes-related lower-limb amputation: evidence from the HRS and ELSA longitudinal cohorts
Source: Front Endocrinol (Lausanne). 2026 Jul 15;17:1874068. doi: 10.3389/fendo.2026.1874068 (PMC13414750; doi:10.3389/fendo.2026.1874068)
Supplement: Supplementary file 1 [file Table1.docx]

Supplementary Table 1. Model selection and performance metrics for GBTM across different cohorts.

| **Model** | **AIC** | **BIC** | **Log-Likelihood** | **Group** | **Sample Size (n)** | **Proportion (%)** | **Average Posterior Probability** |
| --- | --- | --- | --- | --- | --- | --- | --- |
| HRS |  |  |  |  |  |  |  |
| 1-class | 39,062.85 | 39,098.90 | -19,525.43 |  |  |  |  |
| 2-class | 34,218.38 | 34,278.47 | -17,099.19 | 1 | 2252 | 74.89% | 0.922 |
|  |  |  |  | 2 | 755 | 25.11% | 0.438 |
| 3-class | 34,052.80 | 34,136.92 | -17,012.40 | 1 | 160 | 5.32% | 0.916 |
|  |  |  |  | 2 | 2,147 | 71.40% | 0.771 |
|  |  |  |  | 3 | 700 | 23.28% | 0.859 |
| 4-class | 34,060.80 | 34,168.96 | -17,012.40 | 1 | 46 | 1.53% | 0.354 |
|  |  |  |  | 2 | 515 | 17.13% | 0.464 |
|  |  |  |  | 3 | 749 | 24.91% | 0.583 |
|  |  |  |  | 4 | 1697 | 56.43% | 0.899 |
| Elsa |  |  |  |  |  |  |  |
| 1-class | 10,249.70 | 10,278.05 | -5,118.85 |  |  |  |  |
| 2-class | 9,075.62 | 9,122.87 | -4,527.81 | 1 | 479 | 57.50% | 0.537 |
|  |  |  |  | 2 | 354 | 42.50% | 0.537 |
| 3-class | 9,031.20 | 9,097.35 | -4,501.60 | 1 | 85 | 10.20% | 0.754 |
|  |  |  |  | 2 | 612 | 73.50% | 0.654 |
|  |  |  |  | 3 | 136 | 16.30% | 0.548 |
| 4-class | 9,033.43 | 9,118.49 | -4,498.72 | 1 | 102 | 12.24% | 0.318 |
|  |  |  |  | 2 | 203 | 24.37% | 0.326 |
|  |  |  |  | 3 | 473 | 56.78% | 0.387 |
|  |  |  |  | 4 | 55 | 6.60% | 0.343 |
|  | | | |  |  |  |  |
