# Supplementary material for: Associations of activities of daily living and their trajectories with the risk of diabetes-related lower-limb amputation: evidence from the HRS and ELSA longitudinal cohorts
Source: Front Endocrinol (Lausanne). 2026 Jul 15;17:1874068. doi: 10.3389/fendo.2026.1874068 (PMC13414750; doi:10.3389/fendo.2026.1874068)
Supplement: Supplementary file 2 [file Table2.docx]

| Cohort | original variable names | Questionnaire Item | Response Categories | Coding Scheme | Assessment Waves |
| --- | --- | --- | --- | --- | --- |
| HRS | r_amputation amputation | Have you had an amputation because of an injury, disease, or infection? | 1=Yes; 5=No; 8=Don't know; 9=Refused | Yes=1; No=0; DK/Refused=Missing | Follow-up waves after baseline (Wave 9 onward) |
| ELSA | r_amputation amputation | Have you ever had all or part of a leg or foot amputated? | Yes / No | Yes = 1; No = 0 | Follow-up waves after baseline (Wave 4 onward) |

Supplementary Table S2. Definition and Ascertainment of Diabetes-Related Lower-Limb Amputation (DLLA) in HRS and ELSA

Abbreviation: DLLA, diabetes-related lower-limb amputation. Because all participants had physician-diagnosed diabetes at baseline, incident lower-limb amputation occurring during follow-up was defined as DLLA.

The questionnaire items captured any lower-limb amputation, including partial foot or toe amputations where applicable.
