# Supplementary material for: Associations of activities of daily living and their trajectories with the risk of diabetes-related lower-limb amputation: evidence from the HRS and ELSA longitudinal cohorts
Source: Front Endocrinol (Lausanne). 2026 Jul 15;17:1874068. doi: 10.3389/fendo.2026.1874068 (PMC13414750; doi:10.3389/fendo.2026.1874068)
Supplement: Supplementary file 3 [file Table3.docx]

Table S3. Harmonization of Key Variables Across HRS and ELSA

| **Domain** | **HRS** | **ELSA** | **Harmonization Approach** |
| --- | --- | --- | --- |
| ADL | Six ADL items | Six ADL items | Identical scoring (0–6) |
| Diabetes | Self-reported physician diagnosis | Self-reported physician diagnosis | Harmonized definition |
| DLLA | Self-reported lower-limb amputation | Self-reported lower-limb amputation | Harmonized definition |
| Age | Continuous | Continuous | Same coding |
| Sex | Male/Female | Male/Female | Same coding |
| Education | Educational attainment | Educational attainment | Harmonized categories |
| Wealth | Wealth index | Wealth index | Cohort-specific standardization |
| BMI | kg/m² | kg/m² | Same calculation |
| Smoking | Current vs non-current | Current vs non-current | Harmonized categories |
| Drinking | Current vs non-current | Current vs non-current | Harmonized categories |
| Physical activity | Self-reported | Self-reported | Harmonized categories |
| Cancer | Self-reported physician diagnosis | Self-reported physician diagnosis | Harmonized definition |
| Missing data | Complete-case analysis | Complete-case analysis | Same procedure |
| Follow-up design | 3-wave trajectory + subsequent follow-up | 3-wave trajectory + subsequent follow-up | Harmonized framework |
